# Unraveling the Whipple Triad: Non–Islet Cell Tumor–Induced Hypoglycemia

**DOI:** 10.1210/jcemcr/luae006

**Published:** 2024-01-27

**Authors:** Farzahna Mohamed, Frederick J Raal

**Affiliations:** Division of Endocrinology, Department of Internal Medicine, University of the Witwatersrand, Johannesburg, 2193, South Africa; Division of Endocrinology, Department of Internal Medicine, University of the Witwatersrand, Johannesburg, 2193, South Africa

**Keywords:** hypoglycemia, insulin-like growth factor, non–islet cell tumor

## Abstract

Tumor-induced hypoglycemia (TIH) is a rare paraneoplastic phenomenon resulting from several tumor types and mechanisms. Insulinomas are the most common cause of TIH. However, non–islet cell tumors can also trigger hypoglycemia by releasing insulin-like growth factor 2 (IGF-II) or its precursor. We present a case of a 56-year-old woman experiencing spontaneous hypoglycemia due to a pleural-based solitary fibrous tumor. Diagnostic evaluations revealed diminished C-peptide levels, increased IGF-II, and a 4-fold increase in the IGF-II: IGF-I ratio, indicative of non–islet cell tumor hypoglycemia. Localization imaging identified a left pleural mass, confirming the diagnosis. Preoperatively, the patient received intravenous dextrose and corticosteroids, but surgical resection was essential for the resolution of symptoms. The identified tumor, a benign solitary fibrous tumor, was successfully removed, leading to an immediate postoperative cessation of hypoglycemia. Six years post resection, the patient remains symptom free. Managing TIH necessitates an early diagnosis aiming for complete tumor resection, with alternative approaches considered when complete resection is not possible. This case highlights the importance of a systematic diagnostic and management approach for TIH, emphasizing the need to identify the underlying cause, particularly in people without diabetes.

## Introduction

Tumor-induced hypoglycemia (TIH) is a rare paraneoplastic phenomenon resulting from various tumor types and mechanisms, such as excessive insulin secretion predominantly by pancreatic islet β-cell tumors like insulinomas and the very rare non–islet cell tumors that secrete insulin or insulin-like growth factor. Managing non–islet cell tumor hypoglycemia (NICTH) requires a high index of suspicion for an early diagnosis to facilitate a complete resection of the tumor, but alternative medical strategies may be considered when immediate resection is not possible. Cases of TIH resistant to conventional therapy have exhibited positive outcomes with novel somatostatin analogues. The Endocrine Society guideline outlines the assessment and approach to managing hypoglycemia, emphasizing the need to identify the primary cause, particularly in people without diabetes, in whom hypoglycemia may be related to endogenous hyperinsulinism, hormone deficiencies, or non–islet cell tumors. We highlight a case of hypoglycemia secondary to a benign, pleural-based, solitary fibrous tumor.

## Case Presentation

A 56-year-old woman presented with new-onset seizures secondary to severe hypoglycemia. She reported a 4-month history of recurrent episodes of diaphoresis and tremors that were relieved by frequently eating carbohydrates. These episodes occurred in the fasting and postprandial states and had increased in frequency the week before admission, together with being associated with episodes of impaired consciousness. However, despite polyphagia, she reported unintentional weight loss of approximately 8 kg over 12 months. There was no history of diabetes mellitus, alcohol abuse, or exposure to drugs, specifically insulin or sulfonylureas. The examination was unremarkable except for percussion dullness and reduced breath sounds over the left chest wall.

## Diagnostic Assessment

**Figure 1.**
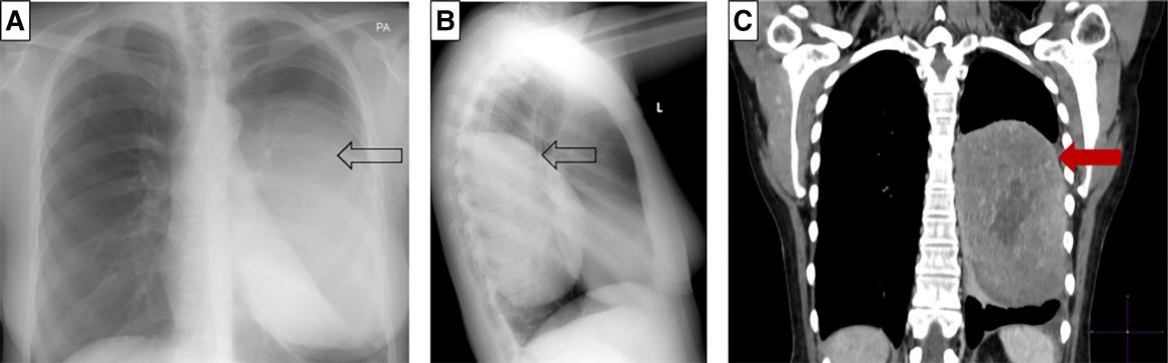
Localization images. A and B, Chest radiograph showing a left pleural–based mass (indicated by arrow): A, posteroanterior; B, lateral. C, Computed tomography of the chest showed a well-delineated, left pleural–based mass measuring 9.7 × 11.8 × 11.8 cm (red arrow).

Investigations during a spontaneous hypoglycemic episode, confirmed with a serum glucose level of 18 mg/dL (1.0 mmol/L), showed an appropriately suppressed C-peptide of 0.03 nmol/L (normal reference range [NR], <0.2; 0.1 μg/L [NR, <0.6 μg/L]); insulin 1.39 pmol/L (NR, <20.8; 0.2 mIU/L [NR, <3 mIU/L]); and β-hydroxybutyrate of 0.2 mmol/L (NR, 0-0.5 mmol/L). The cortisol response was appropriately elevated for the degree of hypoglycemia. Biochemistry was not in keeping with hyperinsulinemia and further investigations were directed at confirming NICTH. The patient’s results were consistent with the diagnosis of NICTH, whereby there was a reduced insulin-like growth factor 1 (IGF-I) level of 5.2 nmol/L (NR, 5.6-22.9 nmol/L), with an elevated insulin-like growth factor 2 (IGF-II) of 220 nmol/L, and a 4-fold increase in the IGF-II:IGF-I ratio of 42.3 (NR < 3) [1‐3]. The diagnosis was further supported by a low IGF binding protein 3 (IGFBP3) of 3.3 mg/L (NR 3.4-6.9; 3.3 μg/mL [NR, 3.4-6.9 μg/mL]) and suppressed growth hormone (GH) of less than 0.1 μg/L (NR, 0.13-9.88 μg/L). Localization images included a chest radiograph that showed an opacification involving the left lower and mid zones (Fig. 1A and 1B). This was further delineated on computed tomography of the chest, showing a well-defined, left pleural–based mass measuring 9.7 × 11.8 × 11.8 cm (Fig. 1C). A primary pleural tumor producing IGF-II was considered the most likely cause of the hypoglycemia.

## Treatment

**Figure 2.**
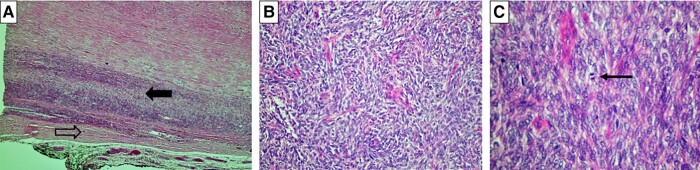
Microscopy photographs, hematoxylin-eosin (H&E) staining. A, Normal pleura (open arrow) with adjacent tumor (solid black arrow) (H&E; 20×). B, Spindle cell neoplasm with a “patternless” growth pattern, comprising tightly packed, round to fusiform cells with indistinct cytoplasmic borders. The cells have regular, round to oval, vesicular nuclei and indiscernible nucleoli. (H&E; 200×). C, Spindle cell neoplasm with 3 mitotic figures (thin black arrow) in 10 high-power fields (H&E; 400×).

**Figure 3.**
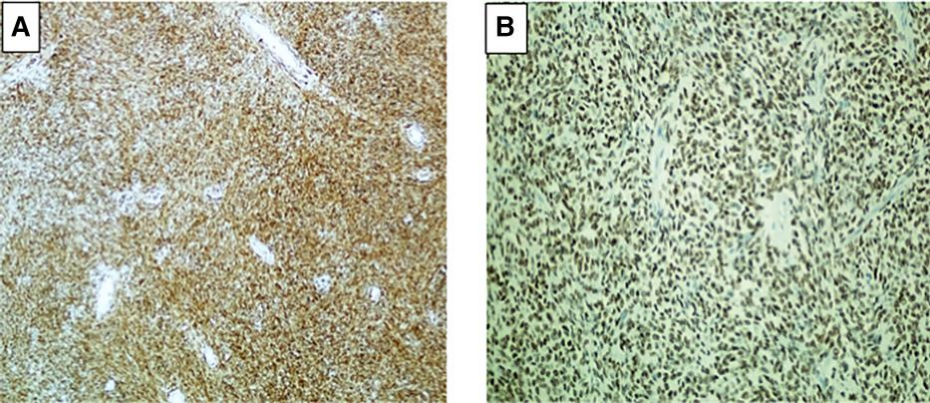
Immunostaining of neoplasm. A, Positive staining with CD 34 (200×). B, Positive nuclear staining with Stat 6 (200×).

Preoperatively, the patient was maintained on a titratable, continuous intravenous infusion of 5% dextrose water, 30 mg prednisone daily, and frequent meals of high carbohydrate content for the management of hypoglycemia. However, the hypoglycemia persisted despite these therapeutic interventions. Surgical resection of the left pleural cavity tumor was performed and macroscopically showed a well circumscribed tumor, measuring 190 mm at its greatest dimension, with a weight of 1060 g. Histopathology revealed spindle-shaped cells with variable atypia and abnormal mitosis with a mitotic index of 3 per 10 high-power fields (Fig. 2A-2C). Immunohistochemistry was positive for CD 34 and Stat 6, in keeping with a solitary fibrous tumor (Fig. 3A and 3B). There were no features suggestive of an invasive malignant tumor.

## Outcome and Follow-up

The patient’s hypoglycemia resolved immediately postoperatively with a 50% reduction in the IGF-II:IGF-I ratio the first day postoperatively. Follow-ups every 6 months for the first 2 years post resection revealed no hypoglycemia or signs of tumor recurrence. The patient is now 6 years post resection and has continued to be well and symptom free.

## Discussion

Hypoglycemia is an important life-threatening emergency to recognize and manage. Fortunately, it is uncommon in people without diabetes. The Endocrine Society recommends further evaluation of hypoglycemia if the Whipple triad is fulfilled [1]. The Whipple triad includes the presence of plasma glucose less than 55 mg/dL (3.0 mmol/L), together with symptoms and signs associated with hypoglycemia, and the resolution of signs after an increase in plasma glucose [1]. It is important to exclude hypoglycemia secondary to medications, either iatrogenic or intentional. A high clinical index of suspicion is necessary to exclude accidental, surreptitious, or malicious use [1]. When the cause of the hypoglycemia is unclear, especially in a healthy individual, a “critical sample” should be conducted during the spontaneous hypoglycemic episode or hypoglycemia should be induced with a 72-hour fast. Plasma glucose, proinsulin, insulin, C-peptide, GH, cortisol, and β-hydroxybutyrate are measured [1]. A plasma glucose response to an intravenous injection of 1.0mg glucagon is also measured. Investigations are directed at differentiating hypoglycemia secondary to hyperinsulinemia (endogenous or exogenous) from various other etiologies, including critical illness, organ failure, and hormone deficiencies, specifically GH and cortisol insufficiency [1, 2].

Endogenous hyperinsulinism is defined as the presence of symptoms, signs, or both, in conjunction with a plasma concentration of glucose less than 55 mg/dL (3.0 mmol/L), inappropriately high serum insulin of 18 pmol/L (>3.0 IU/mL), C-peptide of 0.2 nmol/L (>0.6 μg/L), and proinsulin (>5.0 pmol/L). These are typically indicative of an insulinoma, insulin autoimmune hypoglycemia, or the use of insulin secretagogues, specifically sulfonylureas [1]. Once oral hypoglycemic agents and insulin antibodies are excluded as a cause for hyperinsulinemia, a tumor should be considered as the underlying etiology.

Various mechanisms contribute to TIH. The predominant but rare cause is tumor hyperinsulinism induced by a pancreatic islet β-cell tumor, known as an insulinoma [2]. Localization of an insulinoma can be pursued with various imaging modalities. In the absence of an insulinoma, nesidioblastosis, a condition characterized by diffuse islet cell hyperplasia, should be considered [1].

However, an increasingly recognized source of TIH is NICTH. NICTH causes hypoglycemia predominantly by overproduction of IGF-II or its incompletely processed precursor molecule, big IGF-II [2]. Other mechanisms for NICTH include IGF1 tumor secretion, the generation of autoantibodies targeting insulin or its receptor, secretion of glucagon-like peptide 1 (GLP1), and less commonly from ectopic insulin secretion by a non–islet cell tumor [2]. In patients with advanced cancer, hypoglycemia can occur in the absence of humoral mediators, secondary to increased glucose uptake or reduced glucose output [4, 5]. Nonhormonal factors, including infiltration, damage, or metastases to glucose-regulating organs like the liver, pituitary or adrenal gland, impede glucose output [4, 5]. Furthermore, tumor uptake of glucose and continuous glucose transporter type 4 (GLUT4) expression, especially with a high tumor burden, intensifies glucose consumption [4, 5]. For hypoglycemic patients with concurrent suppression of insulin, proinsulin, C-peptide, and a reduced β-hydroxybutyrate level, the presence of an insulin-like substance should be suspected. This should prompt the assessment of an elevated IGF-I and IGF-II as a cause [6].

Abnormal tumor processing of the IGF-II precursor molecule produces a high-molecular-weight form of IGF-II, termed “big”-IGF-II (10-20 kDa) [6]. This anomaly is associated with irregular IGF-II gene transcription and expression [6]. Big IGF-II is an insulin-like substance that acts via insulin and IGF-I receptors, with a resultant suppression of insulin and GH secretion, as well as a decrease in IGF-I and IGFBP3 [1, 2]. Comparable to insulin, IGF-II triggers hypoglycemia by inhibiting liver glucose output, enhancing skeletal muscle glucose uptake, and suppressing counterregulatory hormones such as glucagon and GH, intensifying susceptibility to hypoglycemia in NICTH [6]. However, IGF-II levels can be low or normal due to assay variations and are thus not a prerequisite in making the diagnosis. Suppressed IGF-I levels lead to an increased IGF-II:IGF-I ratio, serving as a diagnostic indicator for NICTH, but rather an elevated IGF-II:IGFI ratio greater than 3:1 and often exceeding 10:1 is pathognomonic for the diagnosis [2, 6]. GH levels are generally low, together with a β-hydroxybutyrate level of 2.7 mmol/L or less. Furthermore, a plasma glucose increase of at least 25 mg/dL (1.4 mmol/L) after intravenous glucagon indicates insulin-mediated hypoglycemia from IGF [1, 6]. This case was clinically and biochemically in keeping with a NICTH as described earlier.

Once biochemistry is suggestive of NICTH, the next step is the localization of the tumor using cross-sectional imaging of the chest, abdomen, and pelvis, considering that most reported NICTH cases involve tumors in these regions [6]. These tumors are predominantly of mesenchymal origin and include solitary fibrous tumors or mesotheliomas [6]. However, nonmesenchymal tumors, especially hepatocellular carcinoma, can also produce IGF-II [4, 6]. NICTH is commonly referred to as *Doege-Potter syndrome* when associated with fibrous tumors in the thorax. This syndrome, identified for many years, was initially linked to increased glucose utilization by sizable tumors [6]. Despite 70% of these tumors exceeding 10 cm in diameter, the presence of hypoglycemia does not predict the tumor's size or aggressiveness [4].

The primary goal of treatment should be the prompt correction of hypoglycemia, followed by addressing the underlying tumor. Definitive therapy includes complete resection of the tumor, but if the tumor is irresectable, incompletely resected, or has metastasized, stabilization of hypoglycemia can be achieved by increasing gluconeogenesis, inhibiting peripheral uptake of glucose, promoting lipolysis, and increasing glycogenolysis [2, 7]. This can be achieved with the use of either glucocorticoid therapy alone, or in combination with recombinant GH, and long-acting glucagon if there is a positive response to a glucagon stimulation test [2, 6, 7]. Furthermore, glucocorticoids have effectively served as a “bridge” therapy leading up to the resection procedure [6]. However, its use is limited due to the need for continuous dose adjustment, requiring doses beyond a patient-specific threshold, and leading to the recurrence of hypoglycemia on reducing or stopping the glucocorticoid [4].

The prognosis is promising, even in malignant cases, provided a complete resection is successfully performed. In the event of a recurrence, the consideration of local radiotherapy or chemotherapy is an option [6‐8]. Managing TIH poses challenges due to the limited effectiveness and tolerability of currently approved medications, including glucagon infusions, recombinant GH, and corticosteroids, with little success using diazoxide and first-generation somatostatin analogues [4, 5]. If these standard approaches fail to alleviate persistent hypoglycemia in TIH patients, the introduction of pasireotide, a second-generation somatostatin receptor ligand, has demonstrated significant improvement within a month [9]. Pasireotide's enhanced binding affinity for somatostatin receptor (SSTR) subtypes SSTR5 and SSTR2, particularly in insulinomas, contributes to its effectiveness. The mechanism involves pasireotide inducing hyperglycemia by reducing insulin secretion without affecting insulin sensitivity. Due to its robust antihypoglycemic and antitumor properties, pasireotide is considered a logical choice for TIH treatment, though further investigation is necessary [9]. Ongoing research explores therapies such as antibodies against mature and pro–IGF-II as well as anti–IGF-II small interfering RNA for this rare form of hypoglycemia [7].

In conclusion, recognizing the importance of NICTH is crucial in patients with an unclear origin of hypoglycemia. The need for a high clinical suspicion and thorough biochemical evaluation is emphasized for timely NICTH diagnosis. Treatment focuses on promptly correcting hypoglycemia and addressing the underlying tumor, but challenges persist, prompting ongoing research into more effective therapeutic approaches.

## Learning Points

- Despite NICTH being a rare paraneoplastic condition, it is potentially curable, and a high index of suspicion is required for hypoglycemia without a clear etiology.
- NICTH should be suspected when biochemistry indicates reduced insulin, GH, IGF-I, and IGFBP3.
- Elevated IGF-II:IGF-I ratio, often exceeding 10:1, is pathognomonic for NICTH.
- Cross-sectional imaging of the chest, abdomen, and pelvis is crucial for tumor localization.
- Definitive therapy involves complete tumor resection.

## Data Availability

Original data generated and analyzed during this study are included in this published article.

## Abbreviations

GH: growth hormone
GLP1: glucagon-like peptide 1
GLUT4: glucose transporter type 4
H&E: hematoxylin-eosin
IGF-I: insulin-like growth factor 1
IGF-II: insulin-like growth factor 2
IGFBP3: IGF binding protein 3
NICTH: non–islet cell tumor hypoglycemia
NR: normal reference range
SSTR: somatostatin receptor
TIH: tumor-induced hypoglycemia
